# C-Kit Binding Properties of Hesperidin (a Major Component of KMP6) as a Potential Anti-Allergic Agent

**DOI:** 10.1371/journal.pone.0019528

**Published:** 2011-04-29

**Authors:** Hyun-Ja Jeong, Youngjin Choi, Kyu-Yeob Kim, Min-Ho Kim, Hyung-Min Kim

**Affiliations:** 1 Biochip Research Center, Hoseo University, Asan, Republic of Korea; 2 Department of Pharmacology, Kyung Hee University, Seoul, Republic of Korea; 3 High-Enthalpy Plasma Research Center, Chonbuk National University, Jeonju, Republic of Korea; Centre de Recherche Public de la Santé (CRP-Santé), Luxembourg

## Abstract

Accumulation of mast cells can be causally related to several allergic inflammations. Stem cell factor (SCF) as a mast cell chemotaxin induces mast cell migration. To clarify a new effect of Pyeongwee-San extract (KMP6, a drug for indigestion) for the treatment of allergy, we investigated the effects of KMP6 on SCF-induced migration of rat peritoneal mast cells (RPMCs). A molecular docking simulation showed that hesperidin, a major component of KMP6, controls the SCF and c-kit binding by interaction with the active site of the c-kit. KMP6 and hesperidin significantly inhibited SCF-induced migration of RPMCs (*P*<0.05). The ability of the SCF to enhance morphological alteration and F-actin formation was also abolished by treatment with KMP6 or hesperidin. KMP6 and hesperidin inhibited SCF-induced p38 MAPK activation. In addition, SCF-induced inflammatory cytokine production was significantly inhibited by treatment with KMP6 or hesperidin (*P*<0.05). Our results show for the first time that KMP6 potently regulates SCF-induced migration, p38 MAPK activation and inflammatory cytokines production through hindrance of SCF and c-kit binding in RPMCs. Such modulation may have functional consequences during KMP6 treatment, especially mast cell-mediated allergic inflammation disorders.

## Introduction

The mast cell is one of the major effector cells in inflammatory reactions and can be found in most tissues throughout the body [1]. An accumulation of mast cells has been described in several inflammatory conditions, e.g., atopic dermatitis [1], allergic rhinitis [2], asthma [3], and rheumatoid arthritis [4]. Such symptoms require directed migration of mature mast cells or their precursors. Several recent reports provide support for the hypothesis that growth factor and chemokine-mediated chemotaxis of mast cells within tissues can be an important mechanism for a rapid increase in the number of mast cells at sites of inflammation [1], [5].

Stem cell factor (SCF) is a crucial growth factor in mast cell biology. It regulates such diverse cellular functions as proliferation, differentiation, survival, adhesion, and release of inflammatory mediators [6]. SCF acts as a mast cell chemotaxin [1]. Furthermore, injection of SCF into the skin causes mast cell hyperplasia [7], indicating that SCF may be important for the recruitment of mast cell *in vivo*. SCF also induces the pro-inflammatory mediators including histamine, tumor necrosis factor (TNF)-α, interleukin (IL)-1β, IL-6, IL-8, and IL-16 from mast cells [8]. Mast cell-derived TNF-α contributes to allergic reactions through production of an intracellular adhesion molecule (ICAM)-1 [9].

The mitogen-activated protein kinase (MAPK) family comprises at least 6 subsets: extracellular signal-regulated kinase (ERK)1/ERK2, p38 kinase (p38, p38-β, -γ, and -δ), c-JUN NH_2_-terminal protein kinase (JNK), ERK5, ERK6, and ERK7 [10]. MAPKs are believed to play a pivotal role in cell proliferation, apoptosis, differentiation, cytoskeleton remodeling, and cell cycle [11], [12]. SCF similarly activates all MAP kinase [13]. Previously, Sundstrom et al. [14] reported that SCF induced a rapid and transient activation of ERK and p38 in mouse mast cells. Inhibition of p38 activity by SB203580 was paralleled with a marked reduction of migration toward SCF, whereas the effect of the ERK inhibitor was less pronounced.

Pyeongwee-San extract (KMP6) is used for the treatment of gastrointestinal disorders such as inappetance, abdominal distension, borborygmus, diarrhea induced by gastric atony, gastric dilatation, and gastrointestinal catarrh. Many studies have reported that gastrointestinal disorders are closely associated with skin allergic diseases [15], [16]. In this study, we investigated the SCF-dependent effects of KMP6 and its component, hesperidin on migration of rat peritoneal mast cells (RPMCs).

## Methods

### Ethics statement

All protocols were approved by the institutional animal care and use committee of Kyung Hee University [Protocol Number. KHUASP (SE)-09].

### Materials

Avidin peroxidase, metrizamide, SB203580, dimethyl sulfoxide (DMSO), 3-(4, 5-dimethylthiazol-2-yl)-2, 5-diphenyltetrazolium bromide (MTT), and 2′-AZINO-bis (3-ethylbenzithiazoline-6-sulfonic acid) tablets substrates (ABTS) were purchased from Sigma (St. Louis, MO, USA). Recombinant murine SCF, recombinant murine TNF-α and ICAM-1, purified anti-TNF-α and ICAM-1, and biotin-conjugated anti- TNF-α and ICAM-1 were purchased from R&D system (Minneapolis, MN, USA). Fetal bovine serum, α-minimum essential medium (MEM), ampicillin, and streptomycin were purchased from Gibco BRL (Grand Island, NY, USA). Antibody against p38 and phosphorylated-p38 were purchased from Santa Cruz Biotechnology (Santa cruz, CA, USA). *N*-7-nitrobenz-2-oxa-1, 3-diazol-4-phallacidin (NBD-phallacidin) was purchased from Molecular probes (Eugene, Oregon, USA).

### Preparation of KMP6

KMP6 was provided by the Korea Medi Inc. (Seoul, Republic of Korea). We obtained the Pyeongwee-San, HS-PS (an over-the-counter drug for indigestion), from Han Kook Shin Yak pharmaceutical Co. (Nonsan, Republic of Korea) to compare with KMP6. KMP6 is composed of *Atractylodes japonica* Koidzumi (13.3 g), *Magnolia officinale* Rehder et Wils (10 g), *Citrus sunki* Hort. ex Tanaka (10 g), *Zingiber officinale* Roscoe (3.3 g), *Glycyrrhiza uralensis* Fisch (3.3 g), and *Zizyphus jujuba var. inermis* (Bunge) Rehder (6.7 g). The KMP6 was dissolved in distilled water (DW) and filtered with a 0.22 µm syringe filter. HS-PS granules were prepared by dissolving in DW and being autoclaved for the sterilization and kept at 4°C. HS-PS granules (3.5 g) contain some excipients (1.7 g). We made the dose of HS-PS (2 mg/ml) two times stronger than KMP6 (1 mg/ml). Hesperidin is a major constituent of KMP6. KMP6 contained hesperidin of about 5.26 mg/g (data not shown).

### Computational Method

Computer-aided docking simulation was performed by Surflex-Dock (Tripos, St. Louis, MO). The molecular model for the receptor protein, c-kit was obtained from the Protein Data Bank (PDB id 2E9W) with further energy-minimization. The 3D coordinates of each component were prepared by a molecular sketch module. All molecular modeling work was conducted using by a SYBYL X 1.1 package. To obtain an accurate binding mode and affinity data, docking was conducted in the *Geom* mode of Surfelx-Dock. A 6 Å of an expanded search grid, a maximum of 20 conformations per fragment, and a maximum of 100 rotatable bonds per molecule were used as general docking parameters. Spin alignment was activated with a search density of 3 Å and 12 spins per alignment. The docked pose for each component with c-kit was ranked according to Surflex-Dock Score.

### Animals

The original stock of male Wistar rats weighing 200–300 g were purchased from Dae-Han Experimental Animal Center (Taejeon, Chungnam, South Korea). The animals were housed 5–10 per cage in laminar air flow room maintained at 22±1°C and relative humidity of 55±10% throughout the study.

### Preparation of RPMCs

Rats were anesthetized with ether, and injected with 20 ml of Tyrode buffer B (NaCl, glucose, NaHCO_3_, KCL, NaH_2_PO_4_) containing 0.1% gelatin (Sigma) into the peritoneal cavity; the abdomen was gently massaged for about 90 s. The peritoneal cavity was carefully opened, and the fluid containing peritoneal cells was aspirated with Pasteur pipette. Then the peritoneal cells were sedimented at 150 x g for 10 min at room temperature and resuspended in Tyrode buffer B. Mast cells were separated from the major components of rat peritoneal cells (i.e., macrophages and small lymphocytes). In brief, peritoneal cells suspended in 1 ml of Tyrode buffer B were layered onto 2 ml of 0.225 g/ml metrizamide (density 1.120 g/ml; Sigma) and centrifuged at room temperature for 15 min at 400 x g. The cells remaining at the buffer-metrizamide interface were aspirated and discarded; the cells in the pellet were washed and resuspended in 1 ml of Tyrode buffer A (10 mM HEPES, 130 mM NaCl, 5 mM KCl, 1.4 mM CaCl_2_, 1 mM MgCl_2_, 5.6 mM glucose, 0.1% bovine serum albumin) containing calcium. Mast cell preparations were about 95% pure as assessed by toluidine blue staining. More than 97% of the cells were viable as judged by the trypan blue uptake.

### Cell culture

Purified RPMCs were maintained in α-MEM medium (Gibco BRL, USA) with 10% fetal bovine serum (JRH BIOSCIENCE, USA) at 37°C under 5% CO_2_ in air. RPMCs were preincubated with KMP6 (0.01, 0.1, and 1 mg/ml), HS-PS (2 mg/ml), hesperidin (0.01 mg/ml), or dexamethasone (100 nM) at 37°C for 1 before the stimulation with SCF (50 ng/ml) for various times. The cells were separated from the released TNF-α and ICAM-1 by centrifugation at 400 x g for 5 min at 4°C.

### Assessment of cell viability and altered morphology

At time zero and subsequent time-points as indicated, cells were counted in a haemocytometer and viability was assessed by trypan blue dye exclusion. To assess the percentage of cells showing characteristic morphological features, the cells were examined by phase contrast microscopy. Photomicrography was done using Fuji film at ×100 magnification.

### Chemotaxis assay

SCF or the assay medium alone was applied into each well of four-well culture plates. After 10-mm tissue culture inserts (Nalge Nunc International, USA) were placed into each well, RPMCs (500 µl) were added into each insert. The lower compartment of the well was separated from the cell suspension in the upper compartment with an 8 µm pore size polycarbonate membrane of the culture inserts. RPMCs were incubated for 4 h at 37°C in a humidified atmosphere flushed with 5% CO_2_ in air. Following aspiration of nonadherent RPMCs in the upper compartment, cells adherent to the upper surface of the membrane were removed by scraping with a rubber blade. Migrated cells adherent to lower surface of the membrane were fixed with methanol for 5 min and stained with 0.5% toluidine blue. The membranes were mounted on glass slides by routine histological methods. The total number of mast cells that migrated across the membrane was counted under a light microscope.

### MTT assay

To test the cell viability, the MTT colorimetric assay was performed. Briefly, Cells were incubated for 24 h after stimulation in the presence or absence of KMP6, HS-PS, or hesperidin. 50 µl of MTT solution (5 mg/ml) was added, and the cells were incubated at 37°C for an additional 4 h. The crystallized MTT was dissolved in DMSO and the absorbance measured at 540 nm by a microplate reader.

### F-actin formation in RPMCs treated with SCF

Detection of polymerized actin (F-actin) was determined in RPMCs migrating toward the lower side of the membrane according to the method described by Pteiffer and Oliver [17]. Briefly, RPMCs were preincubated with or without KMP6 (1 mg/ml) or hesperidin (0.01 mg/ml) for 1 h and seeded into each culture insert for chemotaxis assay or into each well of 6-well culture plates. After stimulation with SCF for 1 h, RPMCs were fixed with 3% paraformaldehyde/phosphate–buffered saline (PBS) for 1 h at room temperature, washed 3 times with PBS, and permeabilized with 1% Triton X-100/PBS for 15 min. The preparations were stained for 30 min with F-actin specific probe, 1 U/ml NBD-phallacidin at room temperature. All specimens were examined with a confocal laser-scanning microscope using an argon ion laser, which is capable of excitation at 488 nm.

### Enzyme-linked immunosorbent assay (ELISA) of TNF-α and ICAM-1

Sandwich ELISA for TNF-α and ICAM-1 was carried out in duplicate in 96-well ELISA plates (Nunc, USA) coated with each of 100 µl aliquots of anti- TNF-α and ICAM-1 monoclonal antibodies (R&D Systems, Minneapolis, MN, USA) at 1.0 µg/ml in PBS at pH 7.4 and was incubated overnight at 4°C. The plates were washed in PBS containing 0.05% tween-20 (Sigma, St. Lousis, MO, USA) and blocked with PBS containing 1% BSA, 5% sucrose and 0.05% NaN_3_ for 1 h. After additional washes, sample**s** were added and incubated at 37°C for 2 h. Recombinant TNF-α and ICAM-1 were diluted and used as a standard. Serial dilutions starting from 5 ng/ml were used to establish the standard curve. After 2 h incubation at 37°C, the wells were washed and then each of 0.2 µg/ml of biotinylated anti-TNF-α and ICAM-1 were added and again incubated at 37°C for 2 h. After washing the wells, streptavidin-peroxidase was added and plates were incubated for 20 min at 37°C. Wells were again washed and ABTS substrate was added. Color development was measured at 450 nm using an automated microplate ELISA reader. A standard curve was run on each assay plate using recombinant, TNF-α and ICAM-1 in serial dilutions.

### Western blot analysis

Cell extracts were prepared by detergent lysis procedure. Cells were scraped, washed once with PBS, and resuspended in lysis buffer. Samples were vortexed for lysis for a few seconds every 15 minutes at 4°C for 1 h and centrifuged at 15,000 x g for 5 min at 4°C. Supernatants were assayed. Samples were heated at 95°C for 5 min, and briefly cooled on ice. Following the centrifugation at 15,000 x g for 5 min, 50 µl aliquots were resolved by 12% sodium dodecyl sulfate polyacrylamide gel electrophoresis. Resolved proteins were electrotransferred overnight to nitrocellulose membranes in 25 mM Tris, pH 8.5, 200 mM glycine, 20% methanol at 25 V. Blots were blocked for at least 2 h with 1×PBS containing 0.05% tween 20 and 10% nonfat dry milk. The phosphated p38 antibody (1∶500) was added and incubated for 1 h. Afterward, nitrocellulose membrane was washed five times for 15 min with PBST. For protein detection, blot was incubated with anti-mouse secondary antibody conjugated with peroxidase for 40 min, followed by ECL detection.

### Statistical analysis of data

The experiments shown are a summary of the data from at least-three experiments and are presented, as the mean ± S.E.M. Statistical evaluation of the results was performed by independent *t*-test and ANOVA with Tukey post hoc test. The results were considered significant at a value of *P*<0.05.

## Results

### Molecular docking of the components of KMP6 and c-kit interaction

**Figure 1:**
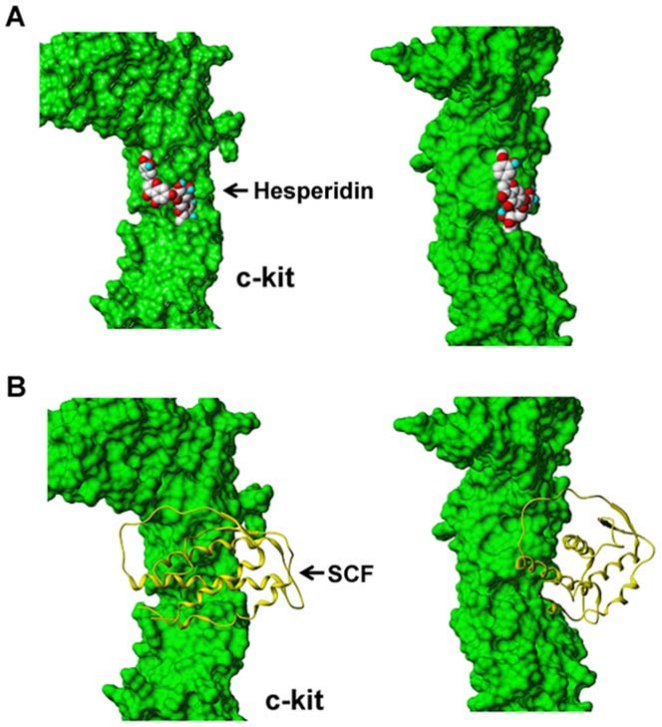
The top view (left) and side view (right) for representative docked poses of the c-kit receptor protein with (A) hesperidin and (B) stem cell factor.

To predict the potential active component in the KMP6, docking simulations were performed using molecular docking software. Table 1 summarizes the final docking score of each component bound to the SCF-binding site of the c-kit receptor protein. The data for docking scores indicated that glycoside compounds including licuraside, hesperidin, and glycyrrhizin were the best components for the c-kit because they had the highest docking score of all the molecules. Hesperidin is a major component of KMP6. In this study, hesperidin was selected for a further evaluation after considering its applicability and attainability. The binding mode of the hesperin for the c-kit was examined in order to compare it with that of SCF which is a natural ligand for this receptor protein (Figure 1).

**Table 1:** Docking scores of the ranked poses for complexes between different components and the c-kit protein.

Component	Docking Score	Origin
Licuraside	7.93	*Glycyrrhiza uralensis* Fisch
Hesperidin	7.50	*Citrus sunki* Hort. ex Tanaka
Glycyrrhizin	7.44	*Glycyrrhiza uralensis* Fisch
Poncirin	7.15	*Citrus sunki* Hort. ex Tanaka
Liquiritin	6.92	*Glycyrrhiza uralensis* Fisch
Magnocurarine	6.48	*Magnolia officinale* Rehder et Wils
Neoisoliquiritin	6.42	*Glycyrrhiza uralensis* Fisch
Honokiol	6.41	*Magnolia officinale* Rehder et Wils
Oleanolic acid	6.31	*Glycyrrhiza uralensis* Fisch
Magnolol	5.51	*Magnolia officinale* Rehder et Wils
Isoliquiritigenin	5.33	*Glycyrrhiza uralensis* Fisch
Eudesmol	5.22	*Magnolia officinale* Rehder et Wils
Hisesol	4.70	*Atractylodes japonica* Koidzumi
Betulic acid	4.63	*Glycyrrhiza uralensis* Fisch
myo-Inositol	4.45	*Citrus sunki* Hort. ex Tanaka
Atractylenenolide III	4.13	*Atractylodes japonica* Koidzumi
Atractylenenolide I	4.07	*Atractylodes japonica* Koidzumi
Acetylatrsctylodinol	3.97	*Atractylodes japonica* Koidzumi
Coumarin	3.34	*Glycyrrhiza uralensis* Fisch
Atractylenenolide II	3.33	*Atractylodes japonica* Koidzumi
Atractylodin	2.68	*Atractylodes japonica* Koidzumi
d-Limonene	2.53	*Citrus sunki* Hort. ex Tanaka

### Effect of KMP6 and hesperidin on SCF-induced RPMCs migration

**Figure 2:**
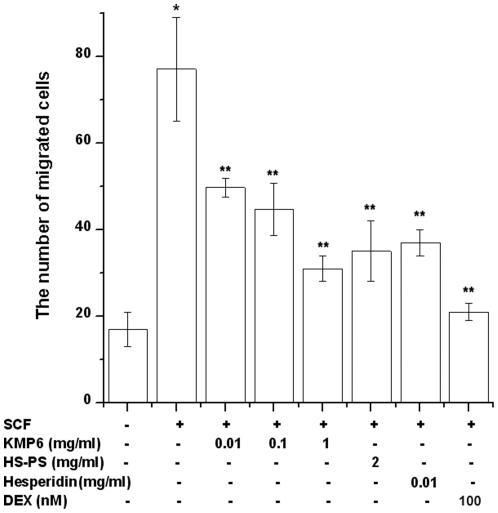
Inhibitory effect of KMP6 and hesperidin on SCF-induced migration. RPMCs (3×10^4^) were treated with KMP6 (0.01, 0.1, and 1 mg/ml), HS-PS (2 mg/ml), hesperidin (0.01 mg/ml), or dexamethasone (100 nM) for 1 h and then stimulated with SCF (50 ng/ml) for 4 h. Migration of RPMCs was assessed by counting the number of RPMCs through the polycarbonate membrane. Each datum represents the mean ± S.E.M. of duplicate determinations from three separate experiments. * *P*<0.05, when compared with the medium alone; ** *P*<0.05, when compared with SCF. DEX, dexamethasone.

The effect of KMP6 and hesperidin on SCF-induced cell migration was determined in a chemotaxis assay using a polycarbonated membrane. SCF (50 ng/ml) was placed in the lower compartment, and then the RPMCs were incubated for 4 h in the upper compartment. SCF significantly increased the number of RPMCs, which migrated toward the lower surface of the polycarbonate membrane through 8-µm pores (*P*<0.05, compared with the medium alone without SCF). This migration was significantly decreased by treatment of KMP6 (0.01, 0.1, and 1 mg/ml), HS-PS (2 mg/ml), hesperidin (0.01 mg/ml), or dexamethasone (100 nM) (*P*<0.05, Figure 2). A treatment of KMP6 resulted in a dose-dependent inhibition of SCF-induced migration. The maximum inhibition occurred at 1 mg/ml. Cell toxicity by KMP6, HS-PS, hesperidin, or dexamethasone was not observed (data not shown).

### Effect of KMP6 and hesperidin on SCF-induced morphological changes

**Figure 3:**
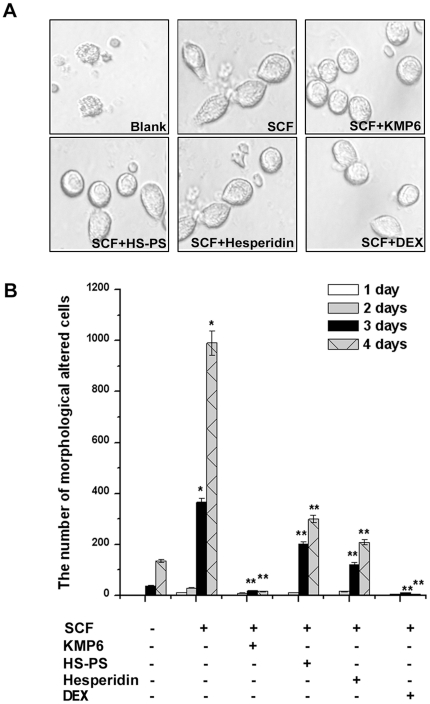
Inhibitory effect of KMP6 and hesperidin on SCF-induced morphological alteration. RPMCs (3×10^4^) were treated with KMP6 (1 mg/ml), HS-PS (2 mg/ml), hesperidin (0.01 mg/ml), or dexamethasone (100 nM) for 1 h and then stimulated with SCF (50 ng/ml) for 4 days. Results are representative of three independent experiments with duplicated samples (A). Morphological alteration was assessed by counting the number of RMPCs for 4 days (B). Each datum represents the mean ± S.E.M. of duplicate determinations from three separate experiments. * *P*<0.05, when compared with the medium alone; ** *P*<0.05, when compared with SCF. Blank, unstimulated cells; DEX, dexamethasone.

Next, we investigated the ability of KMP6 and hesperidin to decrease the morphological change of RPMCs in the presence of SCF. As shown in Figure 3, SCF (50 ng/ml) induced morphological alterations in about 80% of the RPMCs after 4 days of culture. However, the effect of SCF was mostly abolished by treatment with KMP6 (1 mg/ml), HS-PS (2 mg/ml), hesperidin (0.01 mg/ml), or dexamethasone (100 nM).

### Effect of KMP6 and hesperidin on SCF-induced F-actin formation

**Figure 4:**
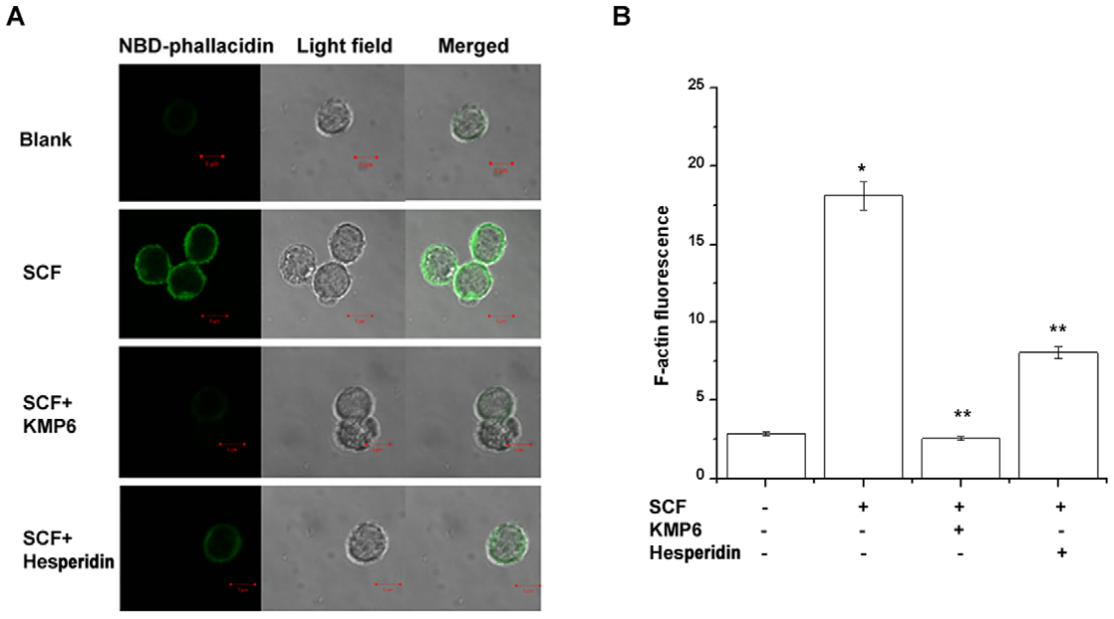
Detection of F-actin in SCF-induced RPMCs with or without KMP6 and hesperidin. RPMCs (3×10^4^) were treated with KMP6 (1 mg/ml) or hesperidin (0.01 mg/ml) for 1 h and then stimulated with SCF (50 ng/ml) for 1 h. Confocal images of RPMCs were stained with NBD-phallacidin. F-actin was visualized using a conforcal laser scanning microscope (A). RPMCs treated with SCF exhibited a high fluorescent intensity (B). Results are representative of three independent experiments with duplicated samples. * *P*<0.05, when compared with the medium alone; ** *P*<0.05, when compared with SCF. Blank, unstimualted cells.

As F-actin formation is well known to be associated with cell motility, we next examined the effect of KMP6 and hesperidin on SCF-induced F-actin formation. F-actin taken from RPMCs that were passing through the pore toward SCF was stained with NBD-phallacidin. Confocal laser scanning microscopic analysis clearly demonstrated that the enhanced formation of F-actin was induced by treatment with 50 ng/ml SCF, but it was markedly blocked by treatment with KMP6 (1 mg/ml) or hesperidin (0.01 mg/ml) (Figure 4A). F-actin levels were obtained from single cell and evaluated as fluorescent intensity (Figure 4B).

### Effect of KMP6 and hesperidin on SCF-induced activation of p38

**Figure 5:**
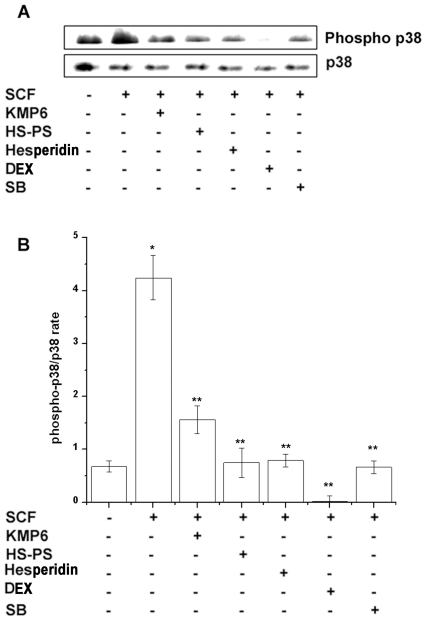
Inhibitory effect of KMP6 and hesperidin on SCF-induced p38 activation. RPMCs (3×10^6^) were treated with KMP6 (1 mg/ml), HS-PS (2 mg/ml), hesperidin (0.01 mg/ml), dexamethasone (100 nM), or SB203580 (20 µM) for 1 h and then stimulated with SCF (50 ng/ml) for 10 min. Total protein was prepared and analyzed for phosphorylated p38 MAPK by Western blotting as described in the experimental procedures (A). Phosphorylated p-38 levels were quantitated by densitometry (B). Results are representative of three independent experiments with duplicated samples. * *P*<0.05, when compared with the medium alone; ** *P*<0.05, when compared with SCF. DEX, dexamethasone; SB, SB203580.

To determine whether the inhibitory action of KMP6 and hesperidin was related to p38 MAPK activation, cell lysates were analyzed for phosphorylated-p38 by immunoblot using an antibody that specifically recognized that phosphorylation form of the protein. Previously, Sundstrom et al. reported that activation of the p38 signaling pathways peaked at 5 to 10 min [14], and the RPMCs were stimulated with SCF for 10 min. As shown in Figure 5, the addition of 50 ng/ml SCF to RPMCs induced phosphorylation of p38 MAPK. KMP6 (1 mg/ml), HS-PS (2 mg/ml), hesperidin (0.01 mg/ml), dexamethasone (100 nM), or SB203580 (20 µM, p38 inhibitor) reduced the levels of phosphorylated-p38 (Figure 5A) in SCF-stimulated RPMCs. The protein levels were quantitated by densitometry (Figure 5B, Pharmacia Biotech, USA).

### Effect of KMP6 and hesperidin on SCF-induced TNF-α and ICAM-1 production

**Figure 6:**
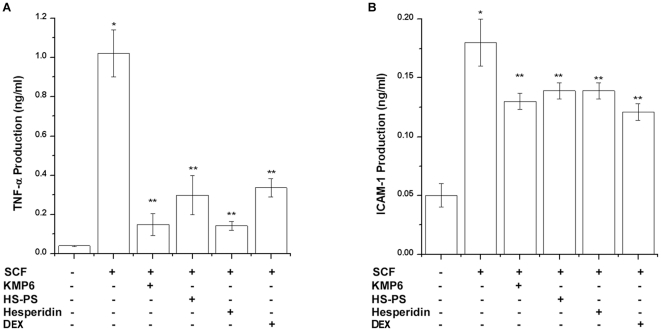
Inhibitory effect of KMP6 and hesperidin on SCF-induced cytokine release from RPMCs. RPMCs (3×10^5^) were treated with KMP6 (1 mg/ml), HS-PS (2 mg/ml), hesperidin (0.01 mg/ml), or dexamethasone (100 nM) for 1 h and then stimulated with SCF (50 ng/ml) for 24 h (TNF-α) or 72 h (ICAM-1). TNF-α (A) and ICAM-1 (B) concentrations were measured from cell supernatants using the ELISA method. Each datum represents the mean ± S.E.M. of duplicate determinations from three independent experiments. * *P*<0.05, when compared with the medium alone, ** *P*<0.05, when compared with SCF. DEX, dexamethasone.

Finally, to determine whether KMP6 and hesperidin can modulate SCF-induced TNF-α and ICAM-1 production from RPMCs, the cells were treated with KMP6 (1 mg/ml), HS-PS (2 mg/ml), hesperidin (0.01 mg/ml), or dexamethasone (100 nM) for 1 h prior to stimulation with SCF for 24 h or 72 h. Culture supernatants were assayed for TNF-α and ICAM-1 protein levels by the ELISA method. As shown in Figure 6A and B, SCF significantly enhanced TNF-α (1.02±0.02 ng/ml, *P*<0.05) and ICAM-1 (0.18±0.04 ng/ml, *P*<0.05) production compared with media control (0.04±0.01 ng/ml for TNF-α and 0.05±0.01 ng/ml for ICAM-1). This induction was significantly inhibited by treatment of KMP6 (1 mg/ml), HS-PS (2 mg/ml), hesperidin (0.01 mg/ml), or dexamethasone (100 nM, *P*<0.05). Inhibition of TNF-α and ICAM-1 production by treatment of KMP6 was about 88.9% and 33.1%, respectively.

## Discussion

In the present study, we showed that KMP6 and hesperidin inhibited SCF-dependent stimulatory effects on migration, morphological alteration, and TNF-α and ICAM-1 production in RPMCs. In addition, KMP6 and hesperidin inhibited SCF-induced p38 MAPK activation.

Directed migration of mast cells towards a chemical gradient of specific chemoattractants locally produced in inflamed tissues is the first integrated event in the process of allergic and non-allergic inflammatory responses [18], [19]. The localization of mast cell precursors to specific tissue sites and the accumulation of mast cells within the given tissue at an inflammatory response were induced by the chemotactic factor, SCF [1]. SCF stimulates specific receptors, c-kit on the cell surface, that initiate several second messenger cascades; this action results in a change in F-actin distribution from azimuthal symmetry around the cell rim to concentration at a particular region involved in migratory behavior [20]. We previously reported that SCF induced morphological alteration and migration of RPMC [21]. Morphological alteration and migration of mast cells by SCF is an important step for the participation in adhesion to tissue [21]. Previously, we also reported that dexamethasone inhibits the migration and F-actin distribution of RPMCs in the presence of recombinant SCF [21]. In this study, we demonstrated that KMP6 and HS-PS inhibited SCF-induced migration of RPMCs and distribution of F-actin. These results including our findings suggest that KMP6 and HS-PS might regulate the migratory process of mast cells following SCF stimulation. Anti-inflammatory, antioxidant, and anti-cancer effects of hesperidin, a main component of *Citrus unshiu*, have been reported [22]–[24]. We reported for the first time that hesperidin reduced SCF-induced mast cell migration and morphological alteration. Therefore, we found that hesperidin is an active compound of KMP6 on SCF-induced mast cell migration.

Binding of SCF to c-kit activates different intracellular signaling components, including the p38 MAPK [25]. MAPK, p38 activation by SCF is of main importance for cell migration toward SCF in general. Suppressing p38 MAPK signaling in mast cells may be a useful tool to reduce mast cell numbers in inflammatory conditions. As described above, KMP6 consists of 6 different herbs. We previously reported that beta-eudesmol, a component of *Atractylodes rhizome* inhibited p38 activation [26]. Other researchers reported that magnolol, a component of *Magnoliae cortex* also inhibited p38 activation [27]. We showed that p38 MAPK activation and activity were blocked when RPMCs were pretreated with KMP6, HS-PS, hesperidin, dexamethasone, or SB203580. Therefore, we suggested that KMP6 reduced the mast cell number via regulation of p38 activation in inflammatory reactions.

TNF-α is constitutively expressed cytokine in mast cells and it is considered a major initiator of inflammation [28]. TNF-α also regulated expression of chemokines such as IL-8, MCP-1, and RANTES. Mucosal inflammation is a feature of both bronchial asthma and allergic rhinitis with evident tissue eosinophilia, mast cells, eosinophils, and T-lymphocytes activation. The initial phase of cell recruitment is the margination and adhesion of leucocytes to the endothelium, prior to their transendothelial migration under a directed chemotactic stimulus. This adhesion occurs through specific ligand-receptor couplets involving leucocyte-endothelial adhesion molecules. One of these cell adhesion molecules is ICAM-1, an important early marker of immune activation and response [29]. Choi et al. reported that hesperidin, a major component of KMP6, inhibited expression of inflammatory cytokines (IL-1beta, IL-6, IL-8, and TNF-α) [22]. Chang et al. reported that glycyrrhetinic acid, a component of *Glycyrrhizae radix*, inhibits ICAM-1 expression via blocking JNK and NF-kappaB pathways in TNF-α-activated cells [30]. We demonstrated that KMP6, HS-PS, and hesperidin inhibited SCF-induced TNF-α and ICAM-1 production. These findings may contribute to understanding the anti-inflammatory effect of KMP6.

Virtual (database) screening (VS) of molecules promises to accelerate the discovery of new drugs and reduce costs by identifying molecules with high probabilities of binding to a target receptor. The large amount of available protein X-ray crystal structures, together with the development of more effective homology modeling techniques, has led recently to a steep increase in docking-based VS studies. This approach needs computational fitting of molecules into a receptor active site using advanced algorithms, followed by the scoring and ranking of these molecules to identify potential leads. In this study, molecular docking results suggested that glycosidic moiety of hesperidin was tightly bound to c-kit in the same manner as the SCF with c-kit. The glycosidc moiety of hesperdin plays a similar role in an α-helical region of the SCF. Furthermore, the flavonoidic backbone of the hesperidin gave an additional affinity with the receptor c-kit protein with aromatic and hydrogen bonding interaction. This ligand bound conformation and docking score of the hesperidin with c-kit provide a molecular-level insight toward explaining its biological efficacy. As described above, hesperidin was also known as a regulator of various intracellular proteins. Taken together, we can presuppose that hesperidin inhibits SCF-induced mast cell migration through regulation of the activity of intracellular proteins and prevention of SCF and c-kit interaction.

In conclusion, we identified a new anti-allergic effect of KMP6. Our results suggest that KMP6 may be useful in the treatment of SCF-mediated inflammatory diseases.
